# Comparative Genomics of Degradative *Novosphingobium* Strains With Special Reference to Microcystin-Degrading *Novosphingobium* sp. THN1

**DOI:** 10.3389/fmicb.2018.02238

**Published:** 2018-09-25

**Authors:** Juanping Wang, Chang Wang, Jionghui Li, Peng Bai, Qi Li, Mengyuan Shen, Renhui Li, Tao Li, Jindong Zhao

**Affiliations:** ^1^State Key Laboratory of Freshwater Ecology and Biotechnology, Institute of Hydrobiology, Chinese Academy of Sciences, Wuhan, China; ^2^University of Chinese Academy of Sciences, Beijing, China; ^3^State Key Laboratory of Protein and Plant Genetic Engineering, College of Life Sciences, Peking University, Beijing, China

**Keywords:** *Novosphingobium*, degradation, comparative genomics, genomic variability, metabolic profile

## Abstract

Bacteria in genus *Novosphingobium* associated with biodegradation of substrates are prevalent in environments such as lakes, soil, sea, wood and sediments. To better understand the characteristics linked to their wide distribution and metabolic versatility, we report the whole genome sequence of *Novosphingobium* sp. THN1, a microcystin-degrading strain previously isolated by Jiang et al. (2011) from cyanobacteria-blooming water samples from Lake Taihu, China. We performed a genomic comparison analysis of *Novosphingobium* sp. THN1 with 21 other degradative *Novosphingobium* strains downloaded from GenBank. Phylogenetic trees were constructed using 16S rRNA genes, core genes, protein-coding sequences, and average nucleotide identity of whole genomes. Orthologous protein analysis showed that the 22 genomes contained 674 core genes and each strain contained a high proportion of distributed genes that are shared by a subset of strains. Inspection of their genomic plasticity revealed a high number of insertion sequence elements and genomic islands that were distributed on both chromosomes and plasmids. We also compared the predicted functional profiles of the *Novosphingobium* protein-coding genes. The flexible genes and all protein-coding genes produced the same heatmap clusters. The COG annotations were used to generate a dendrogram correlated with the compounds degraded. Furthermore, the metabolic profiles predicted from KEGG pathways showed that the majority of genes involved in central carbon metabolism, nitrogen, phosphate, sulfate metabolism, energy metabolism and cell mobility (above 62.5%) are located on chromosomes. Whereas, a great many of genes involved in degradation pathways (21–50%) are located on plasmids. The abundance and distribution of aromatics-degradative mono- and dioxygenases varied among 22 *Novosphingoibum* strains. Comparative analysis of the microcystin-degrading *mlr* gene cluster provided evidence for horizontal acquisition of this cluster. The *Novosphingobium* sp. THN1 genome sequence contained all the functional genes crucial for microcystin degradation and the *mlr* gene cluster shared high sequence similarity (≥85%) with the sequences of other microcystin-degrading genera isolated from cyanobacteria-blooming water. Our results indicate that *Novosphingobium* species have high genomic and functional plasticity, rearranging their genomes according to environment variations and shaping their metabolic profiles by the substrates they are exposed to, to better adapt to their environments.

## Introduction

Hazardous compounds are produced in large quantities by natural and anthropogenic activities. Their release into the environment causes public health concerns. Polycyclic and heterocyclic aromatic compounds have potential toxic, mutagenic, and carcinogenic effects on humans (Dean, 1985; Yoshikawa et al., 1985; Hu et al., 2014; Nguyen et al., 2014; Zhao et al., 2017). For example, organophosphorus insecticide inhibits acetylcholinesterase, which is crucial for the transmission of normal nerve impulses (Jariyal et al., 2018). Microcystins produced by some genera of cyanobacteria inhibit protein phosphatases 1 and 2A and promote tumor formation (Mackintosh et al., 1990; Campos and Vasconcelos, 2010), which causes severe health risks to plants, animals, and humans. Therefore, the elimination of these substances from the environment is of great importance. Microorganisms have evolved efficient degradative pathways for most of these compounds, including bacterial isolates in genera *Rhodococcus, Mycobacterium, Pseudomonas, Sphingomonas*, and *Novosphingobium* (Stolz, 2009).

*Novosphingobium* bacteria are widely distributed and can degrade a wide range of xenobiotic compounds (Tiirola et al., 2005; Hegedus et al., 2017; Kampfer et al., 2018; Sheu et al., 2018). They have been detected in rhizosphere soil (Krishnan et al., 2017), wood (Ohta et al., 2015), contaminated environments (Saxena et al., 2013; Hyeon et al., 2017; Singh et al., 2017), and marine and freshwater environments (Chen et al., 2017; Sheu et al., 2017; Zhang et al., 2017). *Novosphingobium* isolates display a wide variety of metabolic features. For example, *Novosphingobium pokkalii* L3E4^T^, which was isolated from soil of saline-tolerant Pokkali rice, promoted plant growth; *Novosphingobium* sp. MBES04 and *Novosphingobiu*m sp. B-7 isolated from wood degraded lignin-related compounds; and other *Novosphingobiu*m strains from water or solid supports were found to degrade a wide range of xenobiotic aromatic compounds (Tiirola et al., 2002; Hashimoto et al., 2010; Notomista et al., 2011; Choi et al., 2015). A *Novosphingobium* strain isolated from a water sample of Lake Taihu was classified as *Novosphingobium* sp. THN1 (hereafter referred to as THN1) by searching its 16S rRNA sequence against 16S rRNA sequences in GenBank using the BLAST service (Jiang et al., 2011). The isolated strain effectively degraded microcystin-LR (MC-LR), eliminating 91.2% of the MC-LR in the THN1 culture during the first 12 h and completely removing it after 60 h (Jiang et al., 2011).

Although many species in family Sphingomonadaceae, including those in genera *Sphingomonas, Sphingobium*, and *Sphingopyxis*, have been reported to be capable of degrading microcystins (MCs), to our knowledge, THN1 is the only *Novosphingobium* strain found so far that can degrade MCs. Typically, a cluster of four genes (*mlrA, mlrB, mlrC*, and *mlrD*) has been characterized as being responsible for MC degradation (Bourne et al., 1996, 2001). The *mlrA, mlrB*, and *mlrC* genes encode proteins involved in the degradation of MCs, whereas *mlrD* probably encodes a transporter protein. In the first step of the degradation pathway, microcystinase (MlrA) hydrolyzes cyclic MC-LR to form a linear intermediate. Then, MlrB hydrolyzes the linear MC-LR to form a tetrapeptide that is subsequently degraded by MlrC. Recently, two other genes, *mlrE* and *mlrF*, were identified in the genome of *Sphingopyxis* sp. strain C-1 and may be involved in MC degradation in addition to the four genes in the *mlr* cluster (Okano et al., 2015). The *mlrE* and *mlrF* genes were also found in the THN1 genome sequence reported in this study. Comparing the *mlr* gene cluster in different species and strains may help to elucidate how their MC-degrading capacity was acquired.

The genomes of *Novosphingobium* strains isolated from various habitats have been sequenced and characterized, including strains that degrade substrates they have been exposed to. The availability of the genome sequences has facilitated further analysis of the wide distribution and metabolic versatility of *Novosphingobium* strains. In a previous study, the genomes of six marine *Novosphingobium* strains were compared and genes involved in marine adaptation and cell–cell signaling were identified (Gan et al., 2013). Recently, Kumar et al. (2017) identified habitat-specific genes from 27 *Novosphingobium* strains from diverse habitats. Both these studies focused on habitat-specific traits. How these strains evolved their efficient degradative capabilities for the compounds they were exposed to remains to be revealed. Comparative analysis of the genomic contents and functional profiles of *Novosphingobium* strains may throw some light on this issue.

In this study, we sequenced the *Novosphingobium* sp. THN1 genome using a third-generation PacBio RSII system and assembled and annotated the genome sequence. Our aim was to identify potentially important functional genomic characteristics that may facilitate the adaptation and degradation of compounds they were exposed to in the environment.

## Materials and methods

### Genome sequencing, assembly, and annotation

Single-molecule real-time (SMRT) genome sequencing (Gupta, 2008; McCarthy, 2010) of the THN1 genome was performed on a Pacific Biosciences (PacBio) RSII platform (Pacific Biosciences, Menlo Park, CA, USA) by the Annoroad Gene Technology Co. Ltd. (Beijing, China). The *Novosphingobium* strain THN1 previously isolated by Jiang et al. (2011) from a water sample of Lake Taihu was spread onto fresh R2A plates. All plates were incubated at 37°C. After 48-h cultivation, cells were collected and used for genomic DNA extraction. Genomic DNA was extracted using an EZNA Bacterial DNA kit (Omega) according to the manufacturer's instructions. The genomic DNA was sheared using the G-tubes protocol (Covaris, Inc., Woburn, MA, USA). A 20-Kb library was constructed using a PacBio Template Prep kit and sequenced on a PacBio SMRT platform. A total of 916.83 Mb raw data comprising 70,424 reads was generated. *De novo* assembly was performed using the SMRT Analysis pipeline v2.3.0 in conjunction with the HGAP assembler (Chien et al., 2016). Additional assemblies were performed using minimus2 (Treangen et al., 2011). The assembly was validated by aligning the raw reads onto the finished contigs using the Burrows-Wheeler Aligner v0.7.9a (Li and Durbin, 2009). Genes in the assembled sequence were predicted using Prodigal (Hyatt et al., 2010). tRNA, rRNA, and ncRNA were identified by Infernal (Nawrocki et al., 2009) and RNAmmer (Lagesen et al., 2007). Protein-coding sequences were annotated based on BLASTP searches against the downloaded Rfam (Griffiths-Jones et al., 2005), NCBI NR, COG, KEGG (Kanehisa et al., 2008), and Swiss-Prot (Watanabe and Harayama, 2001) databases with an *E*-value cut-off of 1e^−20^ and subsequent filtering for the best hits.

### Phylogenetic analysis

For comparative analysis, we downloaded 21 *Novosphingobium* genome sequences and their annotations (including 5 complete genomes) from the NCBI GenBank database as shown in Table 1. These 21 strains were isolated from different types of ecological niches and can degrade a wide range of natural or xenobiotic compounds, including aromatic hydrocarbons, hexachlorocyclohexane, lignin and thiocyanate. The average chromosome length of the 22 *Novosphingobium* genomes (including THN1) was 5.19 Mb with a range of 3.71–6.92 Mb. Accession numbers of the genomes included in this study and their genomic features are listed in Table 1.

**Table 1:** General features of the 22 genome-sequenced degradative *Novosphingobium* strains used in this study.

**Strain**	**Genome Size (bp)**	**Chromosomes (plasmid)**	**CDS**	**G+C (%)**	**Source of Isolation**	**Compounds degraded**	**NCBI Accession No**.	**References**
*N. aromaticivorans* DSM12444	4,233,314	1(2)	3,928	65.15	River sediments	Aromatic hydrocarbons	NC_007794.1, NC_009426.1, NC_009427.1	Aylward et al., 2013
*Novosphingobium* sp.THN1	4,649,073	1(1)	4,747	63.5	Freshewater lake	Microcystins	CP028347, CP028348	This study
*N. lentum* NBRC107847	4,407,850	N/A	4,020	65.70	Groundwater	Polychlorophenol	BCTW00000000.1	Tiirola et al., 2005
*Novosphingobium* sp. Fuku2-ISO-50	4,422,860	N/A	3,871	63.90	Freshwater lake	Phenol and humic matter	LLZR00000000.1	Hutalle-Schmelzer et al., 2010
*Novosphingobium* sp. B-7	4,909,160	N/A	3,950	64.90	Steeping fluid of eroded bamboo slips	Kraft lignin	APCQ00000000.1	Chen et al., 2012
*Novosphingobium* sp. P6W	6,537,000	N/A	5,611	63.70	Soil sample of plant rhizosphere	Abscisic acid	NZ_CP030352.1, NZ_CP030353.1, NZ_CP030354.1, NZ_CP030355.1	Unpublished data
*N. barchaimii* LL02	5,313,470	N/A	4,835	64.00	HCH-contaminated soil	HCH	JACU00000000.1	Pearce et al., 2015
*N. resinovorum* SA1	6,918,130	1(4)	6,115	64.92	Soil	Sulfanilic acid	NZ_CP017075.1, NZ_CP017076.1, NZ_CP017077.1, NZ_CP017078.1, NZ_CP017079.1	Hegedus et al., 2017
*N. lindaniclasticum* LE124	4,857,930	N/A	4,308	64.60	HCH-contaminated dumpsite	HCH	ATHL00000000.1	Saxena et al., 2013
*N. panipatense* P5:ABC	5,735,120	N/A	4,976	64.70	Noonmati refinery	aliphatics and aromatics	MSQB00000000.1	Unpublished data
*N. pentaromativorans* US6-1	5,457,580	1(5)	4,933	63.01	Marine sediments	Polycyclic aromatic hydrocarbons	NZ_CP009291.1, NZ_CP009294.1, NZ_CP009296.1, NZ_CP009292.1, NZ_CP009293.1, NZ_CP009295.1	Choi et al., 2015
*Novosphingobium* sp. PP1Y	5,313,910	1(3)	4683	63.25	Seawater	Aromatic hydrocarbons	NC_015580.1, NC_015579.1, NC_015583.1, NC_015582.1	D'Argenio et al., 2011
*Novosphingobium* sp. MBES04	5,361,450	N/A	4,287	64.80	Sunken wood	Aromatic compounds	BBNP00000000.1	Ohta et al., 2015
*Novosphingobium* sp. SCN 66-18	4,567,370	N/A	4,061	65.90	Sludge from ponds and wastewater	Thiocyanate	MEGD00000000.1	Kantor et al., 2015
*Novosphingobium* sp. ST904	6,269,460	N/A	4,795	64.50	Rhizosphere	4-nitrophenol, TNT-detoxifying capacities	LGJH00000000.1	Unpublished data
*Novosphingobium* sp. SCN 63-17	5,923,910	N/A	5,114	63.50	Sludge from ponds and wastewater	Thiocyanate	MEGC00000000.1	Kantor et al., 2015
*Novosphingobium* sp. 63-713	5,554,250	N/A	4,822	63.20	Sludge from ponds and wastewater	Thiocyanate	MKVS00000000.1	Kantor et al., 2015
*Novosphingobium* sp. PC22D	5,024,520	N/A	4,550	65.80	Deep-sea water	PAHs	MWMO00000000.1	Unpublished data
*Novosphingobium* sp. Chol11	3,711,410	N/A	3,460	62.30	Freshwater	Steroid	OBMU00000000.1	Yucel et al., 2018
*Novosphingobium* sp. KN65.2	5,204,480	N/A	4,617	63.10	Soil	Carbofuran, including the aromatic moiety	CCBH000000000.1	Nguyen et al., 2015
*N. subterraneum* NBRC 16086	4,700,170	N/A	4,320	63.30	Coastal Plain subsurface sediments	Aromatic compounds	BCZF00000000.1	Balkwill et al., 1997
*N. naphthalenivorans* NBRC 102051	5,236,090	N/A	4,778	63.80	Farmland soil	Naphthalene,dibenzofuran	BCTX00000000.1	Suzuki and Hiraishi, 2007

*CDS, protein-coding DNA sequence; HCH, hexachlorocyclohexane; PAH, polycyclic aromatic hydrocarbons; N/A, not applicable*.

We used two different datasets of representative markers, 16S rRNA sequences and core genes, to construct phylogenetic trees. We performed a multiple sequence alignment of the 16S rRNA sequences from the 22 genomes using Muscle (Edgar, 2004). Unaligned sequences were trimmed from the ends. We used the aligned 16S rRNA sequences to construct a phylogenetic tree using MEGA v7.0.26 (Kumar et al., 2016) with the neighbor-joining method. The robustness of clustering was evaluated by 1000 bootstrap replicates. Phylogeny based on only one common gene may lead to bias; therefore, we used the core genes of the 22 *Novosphingobium* strains to construct another phylogenetic tree with *Sphingobium* sp. YBL2 as an outgroup. The core genes were computed by OrthoMCL v2.0.9 (Li et al., 2003) and retrieved using inhouse scripts. We performed a multiple sequence alignment of the core genes from the 22 genomes using Clustal Omega (Sievers et al., 2011). The end-trimmed core sequences were joined and a phylogenetic tree was constructed using MEGA v7.0.26 with the neighbor-joining method.

We also estimated the phylogenetic relationships based on the whole genomes of 22 *Novosphingobium* strains. Average nucleotide identity (ANI) values were calculated using the MUMmer algorithm of JSpecies v1.2.1 (Kurtz et al., 2004; Richter and Rossello-Mora, 2009; Chan et al., 2012) and visualized as a heatmap using the Morpheus software (https://software.broadinstitute.org/morpheus/). We used CVTree (v3.9.6) (Qi et al., 2004) with a K value of 6 to compute the whole-genome composition vector of the 22 *Novosphingobium* strains. PHYLIP (Retief, 2000) was used to construct a neighbor-joining phylogenetic tree, which was visualized using MEGA v7.0.26 (Kumar et al., 2016).

### Identification of orthologous proteins

Orthologous proteins were detected based on the classification of all encoded proteins in protein families, excluding transposable elements. Groups of orthologous sequences (orthogroups, hereafter referred to as protein families) in all 22 *Novosphingobium* strains were classified by clustering with OrthoMCL v2.0.9 (Li et al., 2003) using a Markov cluster algorithm. Protein families were constructed using the cut-off of 60% percentage identity in the alignments (Snipen and Ussery, 2010) and each protein was assigned to one protein family. The protein families were classified as core, distributed, or unique according to their distribution across the genomes. The core families comprised predicted proteins shared by all 22 strains, the distributed families comprised proteins assigned to a subset of strains, and the unique families comprised proteins assigned to a single strain. The core, distributed, and unique subgroups made up the pan-genome (i.e., the union of all the protein-coding genes present in the 22 *Novosphingobium* genomes; Tettelin et al., 2005). The number and percentage of each family in each strain were calculated for plotting.

### Insertion sequence elements, genomic islands, and crispr detection

Insertion sequences (ISs) were detected by BLAST comparisons (*E*-value ≤ 1e^−5^) against the ISFinder database (Siguier et al., 2006). IslandViewer 4 (Bertelli et al., 2017), which integrates IslandPick (Langille et al., 2008), IslandPathDIMOB (Hsiao et al., 2003), and SIGI-HMM (Waack et al., 2006), were used to predict genomic islands (GIs), as described previously (Zhang et al., 2016). CRISPR arrays were detected using the CRISPRFinder (Grissa et al., 2007) online server to perform BLAST searches against dbCRISPR (CRISPR database). The identified CRISPR arrays were validated as true or false based on whether they were associated with CRISPR associated genes (*Cas*). Only candidate CRISPRs with *Cas* genes in the vicinity were designated as true CRISPRs and selected.

### Functional annotation and metabolic pathway reconstruction

Functional predictions using the COG (Clusters of Orthologous Groups of proteins) database are believed to provide a fast way of describing the functional characteristics of one microbe or a community of microbes (Tatusov et al., 1997) that may be relevant to their function in the environment. The eggNOG database (evolutionary genealogy of genes: Non-supervised Orthologous Groups) contains orthologous groups of genes that were annotated with functional categories derived from COG and KOG (Eukaryotic Orthologous Groups) categories using a graph-based unsupervised clustering algorithm that extended the COG methodology (Jensen et al., 2008). To assess the level of functional diversity among the 22 *Novosphingobium* strains, we assigned the protein-coding genes in the 22 genomes to COG categories by searches against eggNOG v4.5.1 (Huerta-Cepas et al., 2016) using the eggNOG-mapper tool (Huerta-Cepas et al., 2017). The abundance of each COG category was normalized against the average abundance for each strain and used to generate a function heatmap of *Novosphingobium* strains. Core and flexible genes of each strain were extracted and annotated with COG categories as described above for the protein-coding genes.

Predicted proteins in the *Novosphingobium* genomes were also annotated using KAAS (KEGG Automatic Annotation Server) (Moriya et al., 2007). Metabolic pathways were deduced by manual inspection of KEGG Orthology (KO), which was predicted based on comparisons to the KEGG database (Kanehisa and Goto, 2000; Kanehisa et al., 2014).

### Comparative analysis of the microcystin-degrading *mlr* gene cluster

The sphingomonads that belong to family Sphingomonadaceae have been used in bioremediation because of their ability to degrade natural and anthropogenic compounds (Stolz, 2009), an ability that has allowed them to adjust well to contaminated environments. Sphingomonads belong to four genera, *Sphingomonas (sensu strictu), Sphingobium, Novosphingobium*, and *Sphingopyxis* (Takeuchi et al., 2001). A large number of species that can degrade MCs have been isolated in genera *Sphingomonas, Sphingobium*, and *Sphingopyxis*, whereas THN1 is the only known MC-degrading strain in genus *Novosphingobium* and the only one found so far to harbor the MC-degrading *mlr* gene cluster. We suspected that the *mlr* gene cluster in THN1 was horizontally transferred from other MC-degrading species. To test this idea, we retrieved *mlr* gene cluster sequences of species in Sphingomonads from GenBank and compared the *mlr* genes of each pair of microcystin-degrading strains by BLAST alignments. We analyzed the codon usage differences of *mlr* genes and core gens to further clarify this idea. Within the core genes computed in section Identification of orthologous proteins, we selected five genes that are involved in the same COG category with *mlr* gene cluster to analyze codon usage differences. Codon usage of core genes and *mlr* genes was computed and analyzed by graphical codon usage analyser platform (http://gcua.schoedl.de).

## Results and discussion

### Genome sequencing, assembly, and annotation

The THN1 genome was assembled into two contigs: a 3,487,514 bp chromosome with 63.6% GC content and a 1,161,559 bp plasmid with 63.2% GC content (Figure S1). We detected 4747 protein-coding sequences in the assembled sequences, as well as 9 rRNAs, 58 tRNAs, and 9 miscRNAs. Functional analysis based on the COG categories assigned a large number of the protein-coding genes to energy production and conservation (9.39%), amino acid transport and metabolism (9.28%), general function prediction (8.69%), replication, recombination and repair (7.35%), inorganic ion transport and metabolism (6.09%), translation, ribosomal structure and biogenesis and replication (5.94%), and lipid transport and metabolism (5.86%). A number of genes were assigned to the unknown function category (8.69%). Further, the *mlr* gene cluster responsible for MCs degradation was identified in the THN1 genome.

### Phylogenetic analysis of *Novosphingobium* strains and niche adaptation

**Figure 1:**
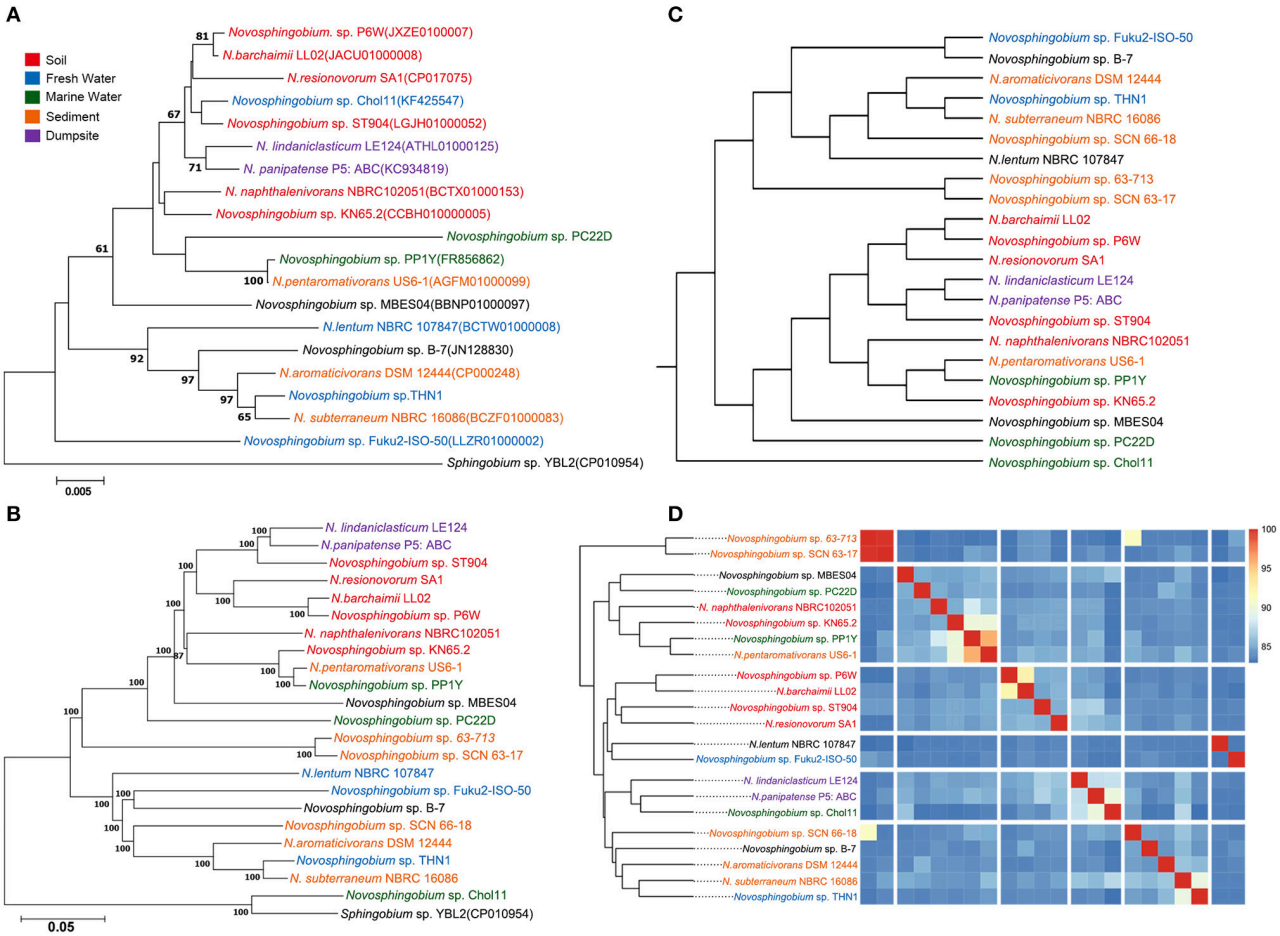
Phylogenetic relationships of 22 *Novosphingobium* strains. Phylogenetic trees based on **(A)** 16S rRNA gene sequences and **(B)** 674 core genes with 1000 bootstraps with *Sphingobium* sp. YBL2 as an outgroup. The bars represent the number of substitutions per nucleotide position. Percentage bootstrap values (≥50%) are shown next to the nodes. **(C)** Whole-genome-based phylogeny trees using a composition vector (CV) approach. **(D)** Average nucleotide identity (ANI)-based phylogenetic dendrograms showing hierarchical clustering of species.

We collected 22 *Novosphingobium* genomes that can degrade substrates. Phylogenetic trees were constructed using 16S rRNAs, core genes, whole-genome composition vectors (CVs), and ANIs (Figure 1). In all four phylogenetic trees, THN1 grouped with *N. subterraneum* NBRC 16086, indicating that THN1 may belong to the species *N. subterraneum*. The topologies of the four phylogenetic trees exhibited some differences. In the phylogenetic tree based on the 16S rRNA sequences, *Novosphingobium* sp. Fuku2-ISO-50 was separated as an individual branch, whereas, in the whole-genome-based CV trees and core gene trees, *Novosphingobium* sp. Chol11 was separated as an individual branch. To confirm the findings from the phylogenetic analysis, we constructed a heatmap based on ANI values. The pairwise ANI values ranged from 82.95% (between THN1 and *Novosphingobium* sp. Chol11) to 95.8% (between *Novosphingobium* sp. SCN63-17 and *Novosphingobium* sp. 63-713). In the phylogenetic trees based on ANIs, *Novosphingobium* sp. 63-713 and *Novosphingobium* sp. SCN63-17 grouped together in a separate clade.

The differences between the whole-genome-based CV and core gene phylogenetic trees revealed that the flexible genes may be important in changing the genome content and shaping the topology of the trees. Several strains in phylogenetic trees clustered together according to their niche specificity. *Novosphingobium* sp. P6W, *N. barchaimii* LL02, and *N. resinovorum* SA1, which were isolated from soil, formed a single clade; *N. lindaniclasticum* LE124, which was isolated from a contaminated dumpsite, clustered with *N. panipatense* P5:ABC from a refinery; and sludge-isolated *Novosphingobium* sp. 63-713 and *Novosphingobium* sp. SCN63-17 clustered together. This result is consistent with the results of an earlier study that found six *Novosphingobium* strains were correlated with their isolation sources (Gan et al., 2013), and in a CV tree of *Acidithiobacillus caldus*, three strains from a copper mine clustered together (Zhang et al., 2016). However, most of the subclades in the phylogenetic trees had more ambiguity in habitat specificity. For example, lake-sourced *Novosphingobium* sp. Fuku2-ISO-50 clustered with *Novosphingobium* sp. B-7 from bamboo slips; and THN1, which was isolated from a lake, clustered with *N. aromaticivorans* DSM12444 from sediment. This result is consistent with previous comparisons of *Novosphingobium* strains from different habitats (Kumar et al., 2017). Further analysis showed that when more strains were included in phylogenetic trees, the trend to cluster according to habitat was more mixed. This suggests that specific environment variations also may influence genome structure (Lin et al., 2013; Ji et al., 2014; Zhang et al., 2016).

### Conserved and variable gene repertoire of *Novosphingobium* genomes

**Figure 2:**
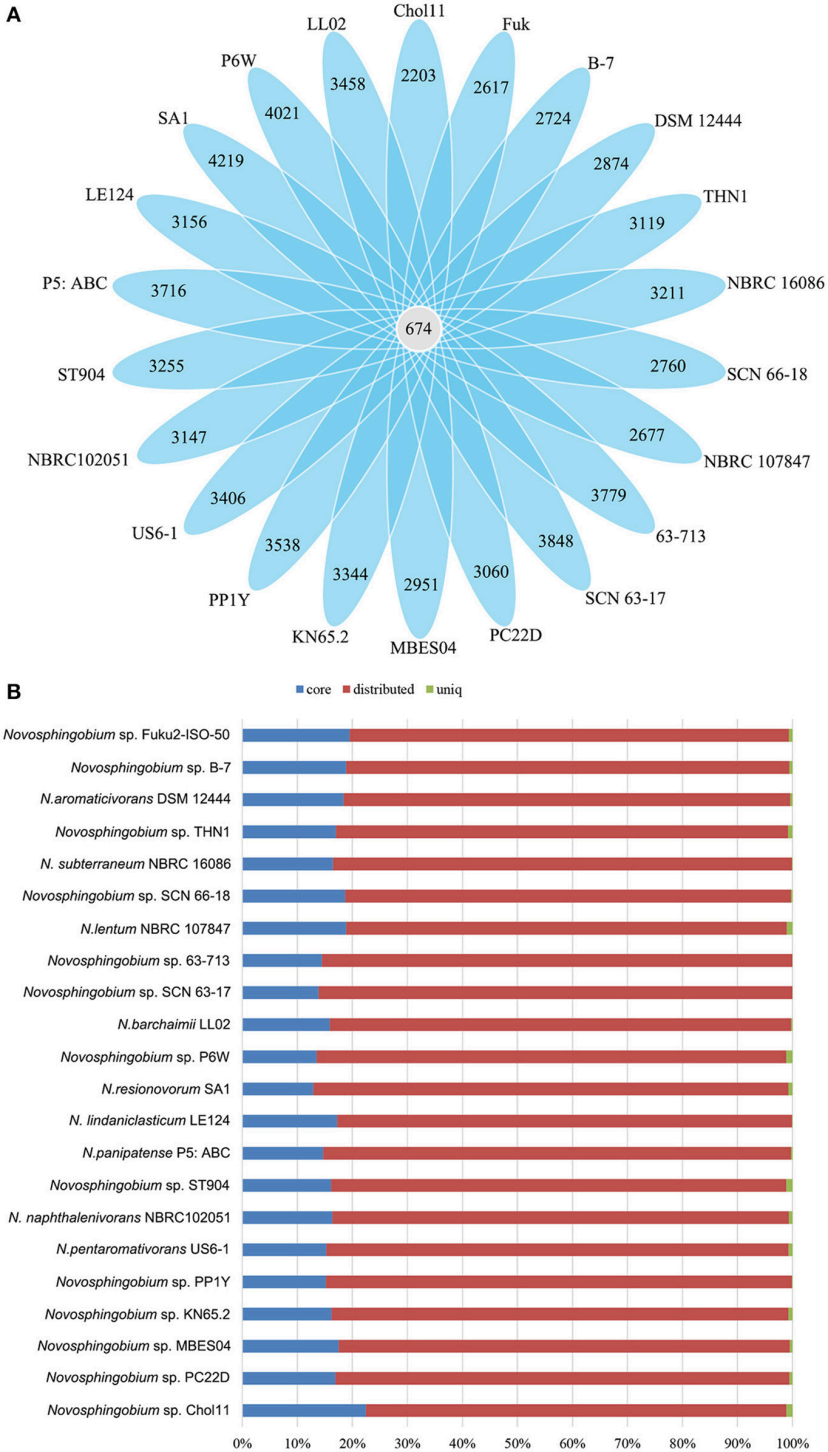
Comparisons of *Novosphingobium* orthologous protein groups in 22 *Novosphingobium* genomes. **(A)** Venn diagram displaying the numbers of core gene families and flexible genes for each of the 22 *Novosphingobium* strains. **(B)** Percentage of core, distributed, and unique genes in each of the 22 genomes.

Pan-genome information indicates the entire genetic potential of a group (Tettelin et al., 2005), where core genes denote conserved functions and flexible genes (or non-core genes) are either unique to an individual genome (unique genes) or shared by a subset of genomes (distributed genes). We carried out pan-genomic studies to investigate the genomic variations among the 22 *Novosphingobium* genomes. Comparative analyses based on groups of orthologous proteins revealed 674 core genes that were shared by the 22 *Novosphingobium* genomes, and over 96% of them were distributed in the chromosome with the remainder in the plasmids (Table 2). The percentages of core genes in each genome varied from 12.89 to 22.49%, which revealed that the core genes made up a small proportion of the total genes in each genome (Figure 2). Therefore, the 22 *Novosphingobium* strains shared a low percentage of common functional proteins. In a previous study, a comparison of six *Novosphingobium* strains revealed a higher number of core orthologous groups (929) (Gan et al., 2013), whereas, in a pan-genomic analysis of 26 sphingomonad genomes, 268 core genes were detected (Aylward et al., 2013). A comparative genomic analysis of 27 habitat-specific Novosphingobium strains showed all 27 strains contained 220 core genes, whereas the genomes of *Novosphingobium* strains from specific habitat shared a higher number of core genes (Kumar et al., 2017). Further analysis indicated that the core genome was asymptotic, and more *Novosphingobium* genomes will result in minor changes in the core genome of *Novosphingobium* (Kumar et al., 2017).

**Table 2:** Size and number of genetic elements and biodegradative enzymes in the six complete *Novosphingobium* genomes.

**Organism**	**Size (kb)**	**No. of:**					
		**Core proteins**	**Unique proteins**	**IS elements**	**GIs**	**Monooxygenases**	**Dioxygenases**
***Novsphingobium aromaticivorans*** **DSM 12444**							
Chromosome	3,561.6	677	12	20	174	7	7
Plasmid pNL1	184.5	1	2	2	5	0	3
Plasmid pNL2	487.3	3	0	1	0	0	2
***Novosphingobium pentaromativorans*** **US6-1**							
Chromosome	3,979.5	683	17	62	94	6	7
Plasmid pLA1	188.5	2	0	32	44	0	2
Plasmid pLA2	62.3	7	1	11	66	0	0
Plasmid pLA3	756.8	0	5	50	57	2	4
Plasmid pLA4	335.7	1	7	13	83	0	0
Plasmid pLA5	134.7	0	2	21	40	0	0
***Novosphingobium*** **sp. PP1Y**							
Chromosome	3,911.5	684	3	67	133	5	11
Plasmid Lpl	192.1	1	0	47	86	0	0
Plasmid Spl	48.7	0	0	2	30	0	0
Plasmid Mpl	1,161.6	4	1	49	86	2	0
***Novosphingobium resinovorum*** **SA1**							
Chromosome	3,778.3	683	9	71	257	6	4
Plasmid pSA1	1,756.8	11	13	74	139	4	9
Plasmid pSA2	960.8	8	12	115	225	2	1
Plasmid pSA3	354.9	5	7	71	265	0	1
Plasmid pSA4	67.3	0	0	10	106	0	0
***Novosphingobium*** **sp. P6W**							
Chromosome1	3,451.3	680	2	41	297	5	1
Chromosome2	2,246.0	17	32	84	163	0	0
Plasmid pP6W1	720.5	4	19	54	95	1	0
Plasmid pP6W2	188.7	2	4	38	89	0	0
***Novosphingobium*** **sp. THN1**							
Chromosome	3,487.5	673	25	112	269	4	5
Plasmid	1,161.6	18	7	108	165	1	2

*IS, insert sequence; GI, genomic island*.

The percentages of unique genes (singletons) in each genome ranged from 0.04 to 1.18% and they were distributed on both the chromosomes (21.95–85.71%) and the plasmids (14.29–78.05%) in six complete genomes (Table 2). The percentages of unique genes on the plasmids were 78.05% for *N. resinovorum* SA1, 40.35% for *Novosphingobium* sp. P6W, 46.88% for *N. pentaromativorans* US6-1, 25% for *Novosphingobium* sp. PP1Y, 21.88% for THN1, and 14.29% for *N. aromaticivorans* DSM12444. The percentage of distributed genes in the each genome varied from 76.43 to 86.36%. Similarly, the alignments of 27 *Streptococcus agalactiae* genomes revealed a great number of distributed genes (72.15%) (Wolf et al., 2018). However, the comparative analyses revealed a relative low proportion of distributed genes in 24 *Shewanella* strains (42.7%), five drug-resistance *Aeromonas hydrophila* strains (14.02–32.75%), 23 *Pasteurella multocida* strains (33.47%) and *Microcystis aeruginosa* strains (38–48%) (Humbert et al., 2013; Hurtado et al., 2018; Zhang et al., 2018; Zhong et al., 2018). The high proportion of flexible genes indicated there was extensive genomic variation among the *Novosphingobium* genomes, which may reflect their variable metabolic profiles. The high percentage of flexible genes and low percentage of unique genes derived from the pan-genome analysis led us to look at their functional classification (section Functional annotation of orthologous groups and comparisons among the *Novosphingobium* strains) for clues into their metabolic diversification.

### Mobile gene elements and crisprs in the *Novosphingobium* genomes

Analysis of the transposable elements predicted by ISFinder indicated that a large number of insertion sequence (IS) elements from various families were distributed over the genomes of the 22 *Novosphingobium* strains (Table S1), indicating these strains had high genomic plasticity to adapt to the various environments. High numbers of IS elements (834, 557, and 487) were identified in the genomes of *N. lindaniclasticum* LE124, N. *naphthalenivorans* NBRC 102051, and *Novosphingobium* sp. ST904, respectively. These elements were distributed on both chromosomes and plasmids and the percentages varied in the six complete genomes (Table 2). A large number of prophage and transposons have been identified previously in the sphingomonads (Aylward et al., 2013). Transposable elements could allow gene exchange between *Novosphingobium* species and other organisms, thereby facilitating their adaption to different habitats.

Besides the ISs, we identified GIs in the genomes of the 22 *Novosphingobium* strains. A large number of these GIs were widespread in the genomes (Table S2), which also suggested high plasticity. Many of the protein-coding sequences in the GIs were annotated as hypothetical proteins. More GIs were identified on chromosomes than on the plasmids in the six complete genomes (Table 2). Previous studies have shown that GIs were relevant to niche-specific adaptation (Wu et al., 2011; Zhang et al., 2016), so the prevalence of GIs in the 22 *Novosphingobium* genomes may imply rapid adaptation occurred to give them a survival advantage in diverse environments.

Surprisingly, our analysis of CRISPRs revealed only a few CRISPR loci in the *Novosphingobium* genomes. One CRISPR array with four spacers was detected in *N. resinovorum* SA1, one with five spacers was detected in *Novosphingobium* sp. B-7, and one with 40 spacers was detected in *Novosphingobium* sp. PC22D, and all three were identified as true CRISPRs. The direct repeat lengths in *N. resinovorum* SA1, *Novosphingobium* sp. B-7, and *Novosphingobium* sp. PC22D were 23, 29, and 29 bp, respectively, and the spacer lengths ranged from 28 bp to 58 bp. The presence of *Cas3* in the vicinity of these three CRISPR arrays indicated that they are type I CRISPR systems. CRISPR is a defense mechanism against bacteriophages and the number of spacers within CRISPR arrays is an indicator of the frequency of viral invasions. Therefore, the low number of spacers in the *N. resinovorum* SA1 and *Novosphingobium* sp. B-7 indicated a low frequency of viral attacks, and the low number of CRISPR arrays reflected vulnerability of the *Novosphingobium* viral defense system.

### Functional annotation of orthologous groups and comparisons among the *Novosphingobium* strains

**Figure 3:**
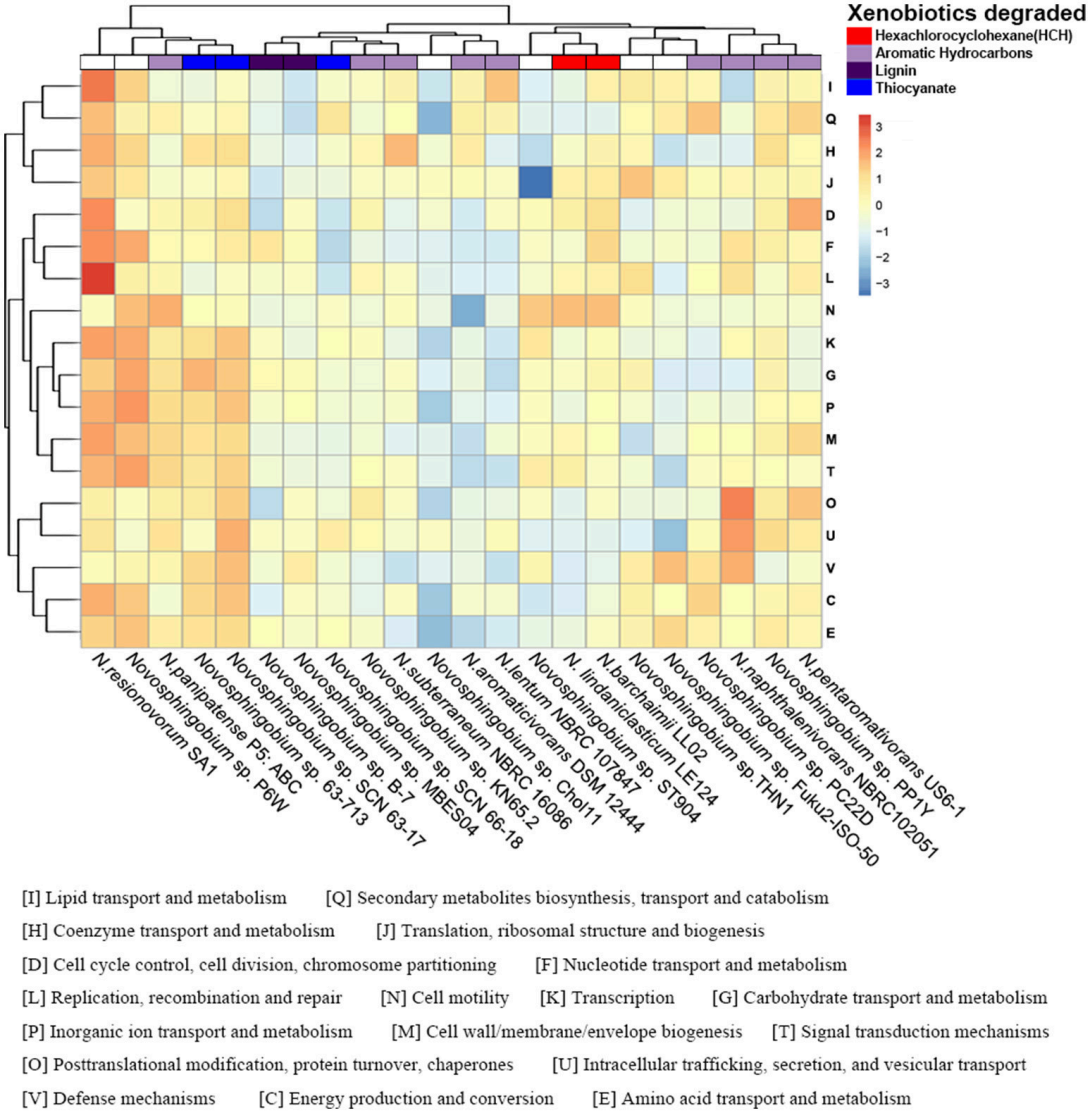
Functional profiling of the *Novosphingobium* genomes. Heatmap showing the normalized relative abundance of the clusters of orthologous groups (COG) categories enriched in the protein-coding genes in the 22 *Novosphingobium* genomes. The strains and COG categories were clustered using the Euclidean distance. The color scale represents the relative abundance of gene content for each category, normalized by sample mean.

COG annotations were assigned to the protein-coding and flexible genes by searches against the eggNOG database. As expected, the annotations of the protein-coding and flexible genes shared the same COG dendrogram. The abundance of annotated COG categories differed among the 22 strains and the difference between two of the clusters in the functional heatmap was significant (*P* < 0.05) (Figure 3). Functional analysis of unique proteins revealed that many COG categories were present in specific species and their abundances were diverse (Figure S2).

The phylogenetic trees of several selected *Novosphingobium* strains were concordant with their habitat specificity, leading us to consider whether the COG functional profiles reflected the habitats or the degradation attributes of the 22 strains. Unlike the phylogenetic clusters, the COG clusters correlated better with the substrate that each strain could degrade than with their environment (Figure 3). This finding suggested a possible link between the functional profiles and substrate-specific traits. For example, *N. barchaimii* LL02, *Novosphingobium* sp. P6W, and *N. resinovorum* SA1 from soil clustered together in the phylogenetic trees but, in the functional heatmap, *N. barchaimii* LL02, which was isolated from a site contaminated with hexachlorocyclohexane (HCH), clustered with *N. lindaniclasticum* LE124, another HCH-degrading strain, and not with *Novosphingobium* sp. P6W and *N. resinovorum* SA1. Consistently, *N. naphthalenivorans* NBRC 102051, *Novosphingobium* sp. PP1Y, and *N. pentaromativorans* US6-1, all with ability to degrade a wide range of aromatic hydrocarbons, clustered into one subgroup, and *Novosphingobium* sp. KN65.2 and *N. subterraneum* NBRC 16086, both of which also degrade aromatic compounds, clustered together. *N. aromaticivorans* DSM12444, which can degrade aromatic compounds, clustered with *N. lentum* NBRC107847, which can degrade chloraromatic compounds. *Novosphingobium* sp. MBES04 and *Novosphingobium* sp. B-7, both of which can degrade lignin, formed one subclade. Thiocyanate-degrading *Novosphingobium* sp. SCN63-17 and *Novosphingobium* sp. 63-713 clustered together. MC-degrading THN1 clustered with *Novosphingobium* sp. Fuk2-ISO-50, which can degrade phenol and humic matter consisting of acetonitrile and H_3_PO_4_. THN1 was isolated from Lake Taihu, China where cyanobacterial bloom breaks out frequently, leading to high concentrations of MCs produced by cyanobacteria in the water (Shen et al., 2003; Duan et al., 2009). To protect itself from damage caused by MC exposure, THN1 has developed the capability of degrading MCs. Similarly, other *Novosphingobium* strains may have rearranged their metabolic profiles to better adapt to specific habitats and to utilize the compounds to which they were exposed.

### Comparison of central metabolism and degradation pathways among the *Novosphingobium* strains

**Figure 4:**
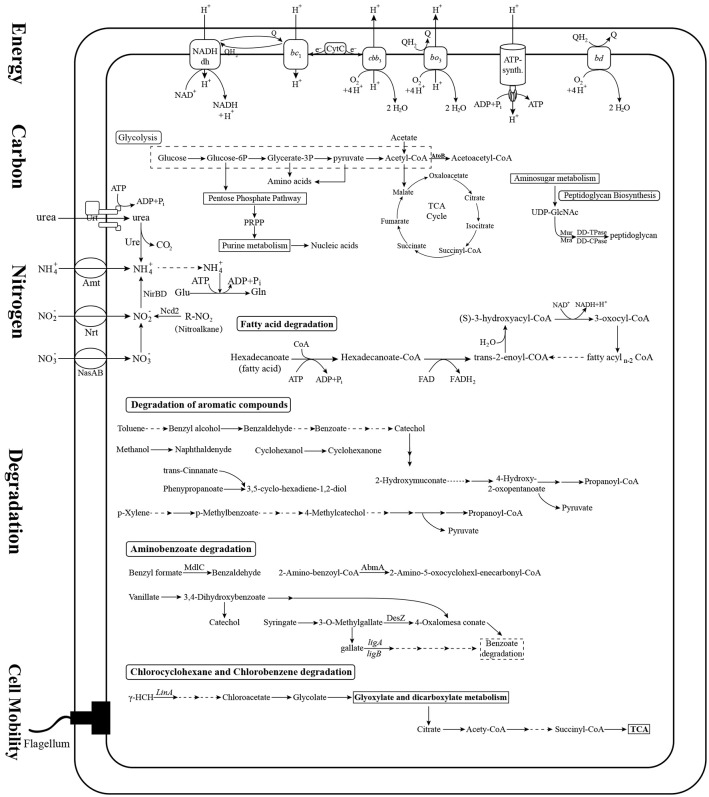
Prediction of the central metabolic potential of 22 *Novosphingobium* strains. Potential metabolic traits associated with carbon, nitrogen, energy metabolism, and degradation pathways were analyzed. The genes predicted to be involved in these metabolic pathways are listed in Table S3.

We assigned metabolic profiles predicted from KEGG pathways to the genes to identify shared metabolic features as well as specific metabolic traits among the 22 *Novosphingobium* strains. In all 22 strains, the genes were assigned to central carbon, nitrogen, energy, cell mobility, and major degradation metabolism pathways (Figure 4, Table S3). All the strains were found to harbor a core set of genes involved in carbon metabolism. They all had a complete glycolysis pathway, pentose phosphate pathway, and tricarboxylic acid cycle, but an incomplete Calvin-Benson-Bassham cycle, lacking genes that encode phosphoribulokinase (*prbK*) and ribulose-bisphosphate carboxylase (*rbcL*/*rbcS*). For nitrogen metabolism, all 22 strains were capable of ammonia uptake via a core set of genes encoding the Amt family of ammonium transporters and transfer ammonia to glutamate using a glutamine synthetase. All strains had genes that could encode assimilatory nitrite reductase (*nirB/nirD*), which reduces nitrite to ammonia, as well as a nitronate monooxygenase encoding gene (*Ncd2*) that converts nitroalkane to nitrite. Furthermore, all 22 strains shared a similar core set of genes that encoded the proteins for uptake and transfer of phosphate and sulfur. The majority of genes involved in central carbon metabolism, nitrogen, phosphate, sulfate metabolism, energy metabolism and cell mobility (above 62.5%) are located on chromosomes. Whereas, a great many of genes involved in degradation pathways (21–50%) are located on plasmids (Table 3). More studies shoud be performed on the intracellular distribution of the genes and their possible exchange/uptake in future.

**Table 3:** Number of genetic elements involved in metabolic pathways in the six complete *Novosphingobium* genomes.

**Organism**	**No. of genes involved in:**				
	**Central carbon metabolism**	**Nitrogen, phosphate and sulfate**	**Energy metabolism**	**Cell mobility and chemotaxis**	**Degradation**
***Novsphingobium aromaticivorans*** **DSM 12444**					
Chromosome	137	29	42	2	78
Plasmid pNL1	0	0	0	0	24
Plasmid pNL2	1	1	0	0	19
***Novosphingobium pentaromativorans*** **US6-1**					
Chromosome	129	25	46	29	59
Plasmid pLA1	0	0	0	0	17
Plasmid pLA2	0	0	0	0	0
Plasmid pLA3	3	14	0	1	24
Plasmid pLA4	0	0	0	0	0
Plasmid pLA5	0	1	0	0	0
***Novosphingobium*** **sp. PP1Y**					
Chromosome	134	25	47	29	86
Plasmid Lpl	0	0	0	0	6
Plasmid Spl	0	0	0	0	0
Plasmid Mpl	3	13	0	0	17
***Novosphingobium resinovorum*** **SA1**					
Chromosome	129	28	43	16	37
Plasmid pSA1	17	4	0	0	33
Plasmid pSA2	0	0	0	3	0
Plasmid pSA3	1	0	4	0	0
Plasmid pSA4	0	0	0	0	0
***Novosphingobium*** **sp. P6W**					
Chromosome1	132	23	42	1	39
Chromosome2	1	5	0	0	31
Plasmid pP6W1	0	0	0	0	8
Plasmid pP6W2	4	0	0	0	0
***Novosphingobium*** **sp. THN1**					
Chromosome	126	23	43	29	63
Plasmid	8	6	0	0	35

Variations in the degradation pathways among these strains were a focus of attention in this study. The 22 *Novosphingobium* strains shared most degradative pathways, including degradation of aromatic compounds, benzoate degradation, and glyoxylate and dicarboxylate metabolism. However, they varied in specific degradative enzymes relevant to the substrate they degrade. The 22 *Novosphingobium* strains contained a large number of genes involved in the degradation of aromatic compounds. We analyzed the encoded mono- and dioxygenases enzymes that catalyze the ring cleavage step critical to aromatic compound degradation, as described previously (Harayama et al., 1992; Aylward et al., 2013). The distribution of genes encoding mono- and dioxygenases varied among the strains (Table S4). For example, *N. resinovorum* SA1 and N. *panipatense* P5:ABC encoded the largest number of monooxygenases and N. *pentaromativorans* US6-1 and *N. resinovorum* SA1 encoded the largest number of dioxygenases. The most abundant monooxygenase family identified was nitronate monooxygenase (NMO, K00459) and the most abundant dioxygenase families included taurine dioxygenase (TauD, K03119) and 4-hydroxyphenylpyruvate dioxygenase (HppD, K00457) (Table S4). Most of the identified genes encoding mono- and dioxygenases were located in the chromosome of *N. aromaticivorans* DSM12444, *Novosphingobium* sp. P6W, *Novosphingobium* sp. PP1Y, *N*. *pentaromativorans* US6-1, and THN1. However, in *N. resinovorum* SA1, more of the dioxygenase-encoding genes were distributed in plasmid pSA1 rather than the chromosome (Table 2). NMO preferentially acts on nitroalkanes, so its presence in all the genomes suggests that all of the strains studied could be involved in the utilization of nitroalkane. Our central metabolic profile also revealed that the 22 Novosphingobium strains could metabolize nitroalkane to nitrite in nitrogen metabolic pathways.

The degradation pathway mediated by the *lin* genes has been associated with HCH degradation (Pearce et al., 2015). The initial steps in the degradation of HCH involve dehydrochlorination mediated by LinA or hydrolytic dechlorinations mediated by LinB, followed by LinC-catalyzed dehydrogenation (Nagata et al., 1993, 1994; Trantirek et al., 2001). The downstream pathway involves reductive dechlorination by the glutathione S-transferase LinD, followed by ring cleavage and conversion by LinE and LinF, respectively (Miyauchi et al., 1998, 1999). However, a growing number of newly sequenced strains have been found to have missing key *lin* components of the pathway (Dogra et al., 2004; Kaur et al., 2013; Kohli et al., 2013; Kumar Singh et al., 2013; Mukherjee et al., 2013). In our analysis of the HCH degradation pathway, *linA* was present only in *N. barchaimii* LL02 but absent in another HCH-degrading strain *N. lindaniclasticum* LE124. Because *N. lindaniclasticum* LE124 was confirmed to be able to degrade HCH (Saxena et al., 2013), the initial degradation-related gene remains to be characterized in this strain. *linB* was present in *N. resinovorum* SA1, *Novosphingobium* sp. SCN 66-18, and *Novosphingobium* sp. Chol11, suggesting that these strains could degrade HCH. linC was identified in *N. lindaniclasticum* LE124, *N. panipatense* P5:ABC, *N. pentaromativorans* US6-1, and *Novosphingobium* sp. ST904. *linD–F* were absent in the genomes of the 22 strains. Previous genomic comparisons of Sphingomonadaceae strains also found considerable variations in the presence of *lin* genes (*linA*–*F*) among HCH-degrading bacteria and it was speculated that the absence of some genes may reflect early stages in the acquisition of the HCH-degrading pathway (Pearce et al., 2015).

Overall, the metabolic analysis revealed that the 22 strains shared genes involved in central carbon, nitrogen, energy metabolism, and cell mobility, but varied in specific degradation enzymes and pathways, which may be related to the substrates they were exposed to.

### Comparative analysis of the Mc-degrading *mlr* gene cluster in THN1 and other strains in family *Sphingomonadaceae*

**Figure 5:**
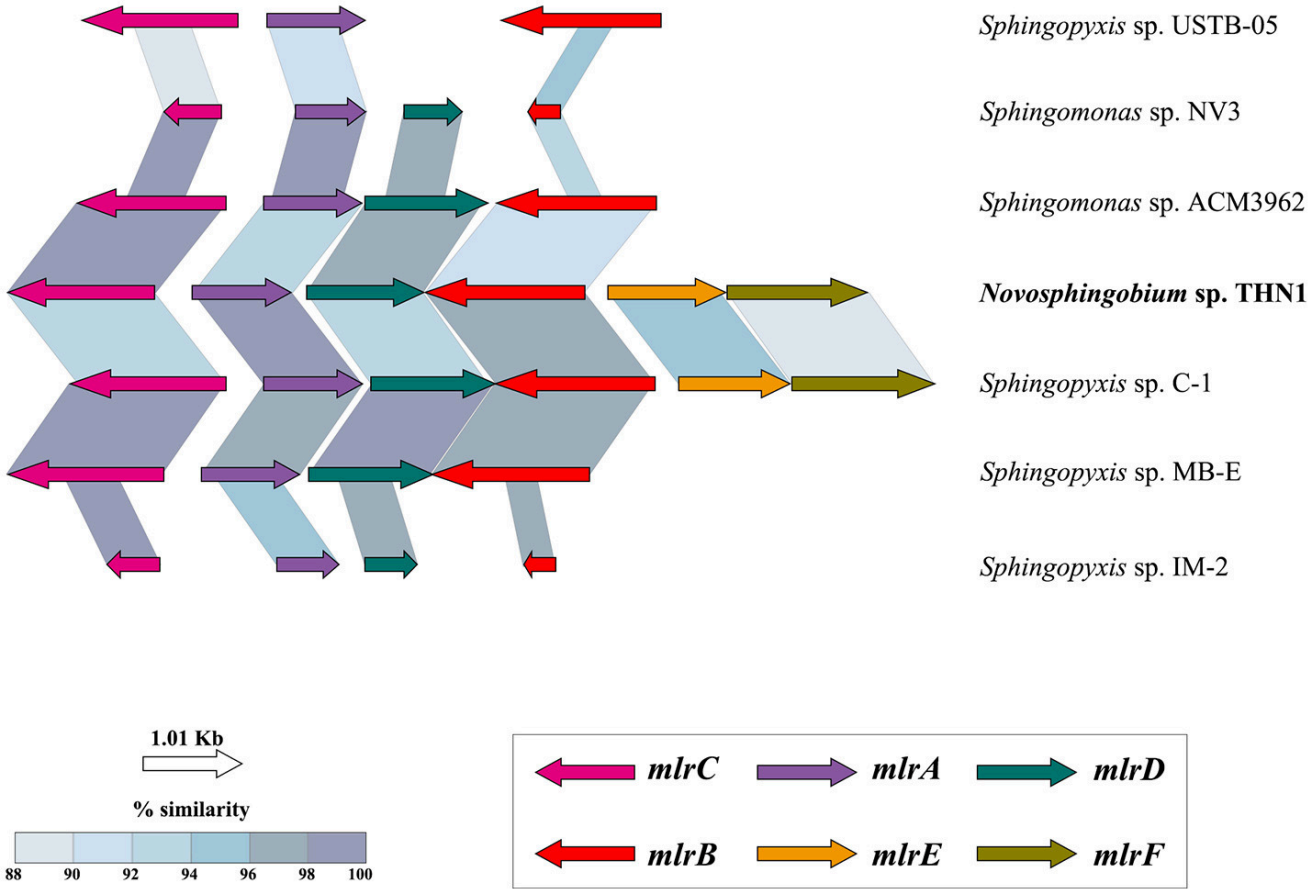
Structure and similarity of the *mlr* gene cluster in THN1 and other species in family Sphingomonadaceae. TBLASTX was used to identify the *mlr* sequences and to determine similarities and alignment lengths. *mlrA, mlrB*, and *mlrC* encode proteins involved in the degradation of microcystins, and *mlrD* encodes a transporter protein. The functions of *mlrE* and *mlrF* are unknown.

THN1 was isolated from a water sample of Lake Taihu with high concentrations of MCs (Jiang et al., 2011). The THN1 genome contained all the functional genes for MC degradation (section Genome sequencing, assembly, and annotation). We compared the *mlr* cluster in THN1 with that in other MC-degrading strains from other genera to investigate the acquisition of *mlr* genes and the capacity of THN1 to degrade MCs. The *mlr* cluster was largely syntenic across all the strains tested with the same organization and with gene sequence identity ≥85% (Figure 5). Among these strains, the whole genome of *Sphingopyxis* sp. C-1 was sequenced and thus the *mlr* genes in the genome showed the highest coverage with the *mlr* genes of THN1. Whereas the *mlr* genes in *Sphingomonas* sp. ACM3962, *Sphingomonas* sp. NV3, *Sphingopyxis* sp. USTB-05, *Sphingopyxis* sp. MB-E, and *Sphingopyxis* sp. IM-2 were obtained by PCR amplifications and were incomplete and thus showed low coverage with the *mlr* genes of THN1. MC-degradation activity is prevalent in cyanobacteria blooming environments where the concentration of MCs is high. All the strains with a characterized *mlr* gene cluster in Figure 5, including THN1, were isolated from lakes or reservoirs with cyanobacterial blooms and high concentrations of MCs (Jones et al., 1994; Okano et al., 2009; Wang et al., 2010; Somdee et al., 2013; Lezcano et al., 2016; Maghsoudi et al., 2016). So far, no other *Novosphingobium* isolates have been found to possess *mlr* genes and the capability of degrading MCs; therefore, the *mlr* gene cluster in THN1 may have been obtained from other bacterial genera in the same niche by horizontal gene transfer. The codon usage differences between core genes and *mlr* genes of THN1 may explain this inference (Figure S3). The acquisition of the MC-degradation ability guaranteed the survival of this *Novosphingobium* strain. *Novosphingobium* species can rearrange their genomes and functional profiles to adapt to local environments, which may explain their high survival rates and distribution diversity.

## Conclusions

Orthologous protein analysis revealed the presence of a large number of flexible genes and mobile gene elements in 22 *Novosphingobium* strains, suggesting these strains had high plasticity of genomic contents. Analysis of the COG functional profiles showed that the COG clusters correlated with the substrate that each strain could degrade. Further, the metabolic profiles predicted from the KEGG pathways analysis showed that although all 22 strains shared genes involved in central carbon, nitrogen, phosphate, sulfate, energy metabolism, and cell mobility pathways, specific degradative enzymes and reactions varied. Thus, we propose that environment variation shaped the general genome structure of these *Novosphingobium* strains as a result of the compounds they were exposed to and redirected the degradation profile to facilitate beneficial substrate utilization. For example, to degrade the high concentration of MCs in Lake Taihu, THN1 may have acquired a *mlr* gene cluster from other genera, which was integrated into the genome. These genome-guided findings, to some extent, enhance current knowledge of the genomic and metabolic diversity of *Novosphingobium* species.

## Data availability

The raw PacBio sequence of the *Novosphingobium* sp. THN1 genome has been deposited in the Sequence Read Archive of the National Center for Biotechnology Information under accession number SRP143911. The complete chromosome and plasmid genome sequences have been deposited in DDBJ/ENA/GenBank under accession numbers CP028347 and CP028348, respectively. Source codes, example softwares and genomic data are available at https://github.com/shenmengyuan/Novo_comparison.

## Author contributions

JW carried out the data analysis and prepared the manuscript draft. TL, RL, and JZ helped design the project and revised the manuscript. CW and JL assisted in formatting figures. PB and MS helped revised the manuscript. QL assisted in data analysis. All authors discussed the manuscript draft and agreed to the final content.

### Conflict of interest statement

The authors declare that the research was conducted in the absence of any commercial or financial relationships that could be construed as a potential conflict of interest.
