# Supplementary material for: Comparative Genomics of Degradative Novosphingobium Strains With Special Reference to Microcystin-Degrading Novosphingobium sp. THN1
Source: Front Microbiol. 2018 Sep 25;9:2238. doi: 10.3389/fmicb.2018.02238 (PMC6167471; doi:10.3389/fmicb.2018.02238)
Supplement: Supplementary file 1 [file Table_1.DOCX]

**Supplementary Table S1** Classification of insert sequences (IS) families in the *Novosphingobium* strains.

| **IS Family** | **Number** | | | | | | | | | | | | |
| --- | --- | --- | --- | --- | --- | --- | --- | --- | --- | --- | --- | --- | --- |
|  | **SA1** | **US6-1** | **LE124** | **THN1** | **PP1Y** | **P6W** | **P5:ABC** | **NBRC107847** | **LL02** | **DSM12444** | **MBES04** | **Fuk** | **B-7** |
| IS110 | 21 | 19 | 455 | 37 | 46 | 37 | 120 | 29 | 153 | 4 | 52 | 34 | 35 |
| IS1182 | 6 | 0 | 0 | 0 | 18 | 19 | 0 | 9 | 11 | 0 | 22 | 17 | 33 |
| IS1380 | 20 | 68 | 146 | 91 | 28 | 67 | 21 | 57 | 35 | 0 | 95 | 13 | 106 |
| IS1595 | 11 | 30 | 79 | 55 | 12 | 26 | 29 | 13 | 34 | 13 | 63 | 16 | 50 |
| IS21 | 2 | 5 | 14 | 7 | 15 | 6 | 21 | 0 | 11 | 0 | 17 | 17 | 9 |
| IS256 | 1 | 13 | 10 | 2 | 5 | 6 | 9 | 9 | 36 | 1 | 18 | 1 | 12 |
| IS3 | 5 | 12 | 33 | 14 | 8 | 22 | 25 | 2 | 21 | 0 | 34 | 0 | 31 |
| IS30 | 0 | 8 | 37 | 2 | 0 | 2 | 13 | 0 | 7 | 0 | 16 | 8 | 9 |
| IS481 | 0 | 1 | 0 | 0 | 0 | 2 | 0 | 0 | 2 | 0 | 2 | 0 | 1 |
| IS481 | 0 | 0 | 8 | 0 | 0 | 2 | 9 | 0 | 1 | 0 | 5 | 2 | 3 |
| IS5 | 0 | 2 | 1 | 0 | 1 | 3 | 1 | 0 | 0 | 0 | 0 | 7 | 2 |
| IS6 | 0 | 7 | 0 | 0 | 0 | 0 | 0 | 0 | 0 | 0 | 0 | 2 | 0 |
| IS630 | 0 | 0 | 0 | 1 | 3 | 6 | 0 | 0 | 4 | 0 | 0 | 12 | 0 |
| IS66 | 0 | 0 | 2 | 0 | 10 | 0 | 5 | 0 | 0 | 0 | 8 | 4 | 0 |
| IS701 | 1 | 1 | 18 | 4 | 3 | 26 | 20 | 0 | 2 | 0 | 23 | 14 | 0 |
| IS91 | 0 | 0 | 1 | 0 | 0 | 0 | 1 | 0 | 0 | 0 | 1 | 12 | 0 |
| IS91 | 0 | 16 | 13 | 0 | 9 | 0 | 0 | 0 | 10 | 0 | 27 | 0 | 0 |
| ISKra4 | 0 | 0 | 17 | 0 | 3 | 0 | 0 | 0 | 5 | 0 | 0 | 0 | 0 |
| Total | 70 | 184 | 834 | 213 | 161 | 228 | 274 | 119 | 342 | 18 | 383 | 159 | 291 |

| **IS Family** | **Number** | | | | | | | | |
| --- | --- | --- | --- | --- | --- | --- | --- | --- | --- |
|  | **SCN63-17** | **SCN66-18** | **63-713** | **Chol11** | **ST904** | **KN65.2** | **PC22D** | **NBRC16086** | **NBRC102051** |
| IS110 | 2 | 3 | 1 | 2 | 25 | 4 | 13 | 17 | 16 |
| IS1182 | 6 | 0 | 7 | 0 | 1 | 2 | 0 | 6 | 4 |
| IS1380 | 10 | 2 | 13 | 0 | 5 | 13 | 0 | 0 | 17 |
| IS1595 | 6 | 3 | 0 | 1 | 1 | 0 | 10 | 1 | 8 |
| IS21 | 11 | 8 | 50 | 0 | 152 | 43 | 45 | 8 | 193 |
| IS256 | 27 | 0 | 17 | 0 | 0 | 0 | 41 | 0 | 44 |
| IS3 | 31 | 10 | 42 | 7 | 124 | 61 | 114 | 58 | 90 |
| IS30 | 0 | 0 | 0 | 0 | 5 | 1 | 0 | 0 | 0 |
| IS481 | 0 | 0 | 0 | 0 | 0 | 0 | 10 | 3 | 0 |
| IS5 | 5 | 7 | 9 | 3 | 81 | 56 | 73 | 29 | 113 |
| IS6 | 1 | 2 | 1 | 0 | 12 | 18 | 0 | 0 | 3 |
| IS630 | 1 | 0 | 1 | 0 | 16 | 1 | 5 | 1 | 8 |
| IS66 | 2 | 0 | 1 | 1 | 9 | 22 | 0 | 12 | 30 |
| IS701 | 4 | 0 | 0 | 0 | 1 | 15 | 7 | 8 | 15 |
| IS91 | 0 | 1 | 0 | 0 | 2 | 2 | 3 | 7 | 2 |
| ISL3 | 2 | 1 | 1 | 0 | 1 | 1 | 0 | 0 | 0 |
| ISAzo13 | 0 | 7 | 0 | 0 | 0 | 0 | 0 | 0 | 0 |
| ISNCY | 0 | 0 | 0 | 0 | 0 | 2 | 0 | 0 | 0 |
| Tn3 | 19 | 9 | 13 | 5 | 52 | 12 | 10 | 15 | 14 |
| Total | 127 | 53 | 156 | 19 | 487 | 253 | 331 | 165 | 557 |
