# Supplementary material for: Comparative Genomics of Degradative Novosphingobium Strains With Special Reference to Microcystin-Degrading Novosphingobium sp. THN1
Source: Front Microbiol. 2018 Sep 25;9:2238. doi: 10.3389/fmicb.2018.02238 (PMC6167471; doi:10.3389/fmicb.2018.02238)
Supplement: Supplementary file 5 [file Data_Sheet_1.PDF]

Supplementary Material

Comparative genomics of degradative *Novosphingobium* strains with special reference to the microcystin-degrading *Novosphingobium* sp. THN1

Juanping Wang[1, 2†], Chang Wang[1, 2†], Jionghui Li[1, 2], Peng Bai[1, 2], Qi Li[1, 2], Mengyuan Shen[1, 2], Renhui Li[1*], Tao Li[1*], Jindong Zhao[1]

*** Correspondence:** Tao Li: litao@ihb.ac.cn; Renhui Li: reli@ihb.ac.cn

[†]These authors have contributed equally to this work.

File content

This file contains **Supplementary Figures S1-S3**.

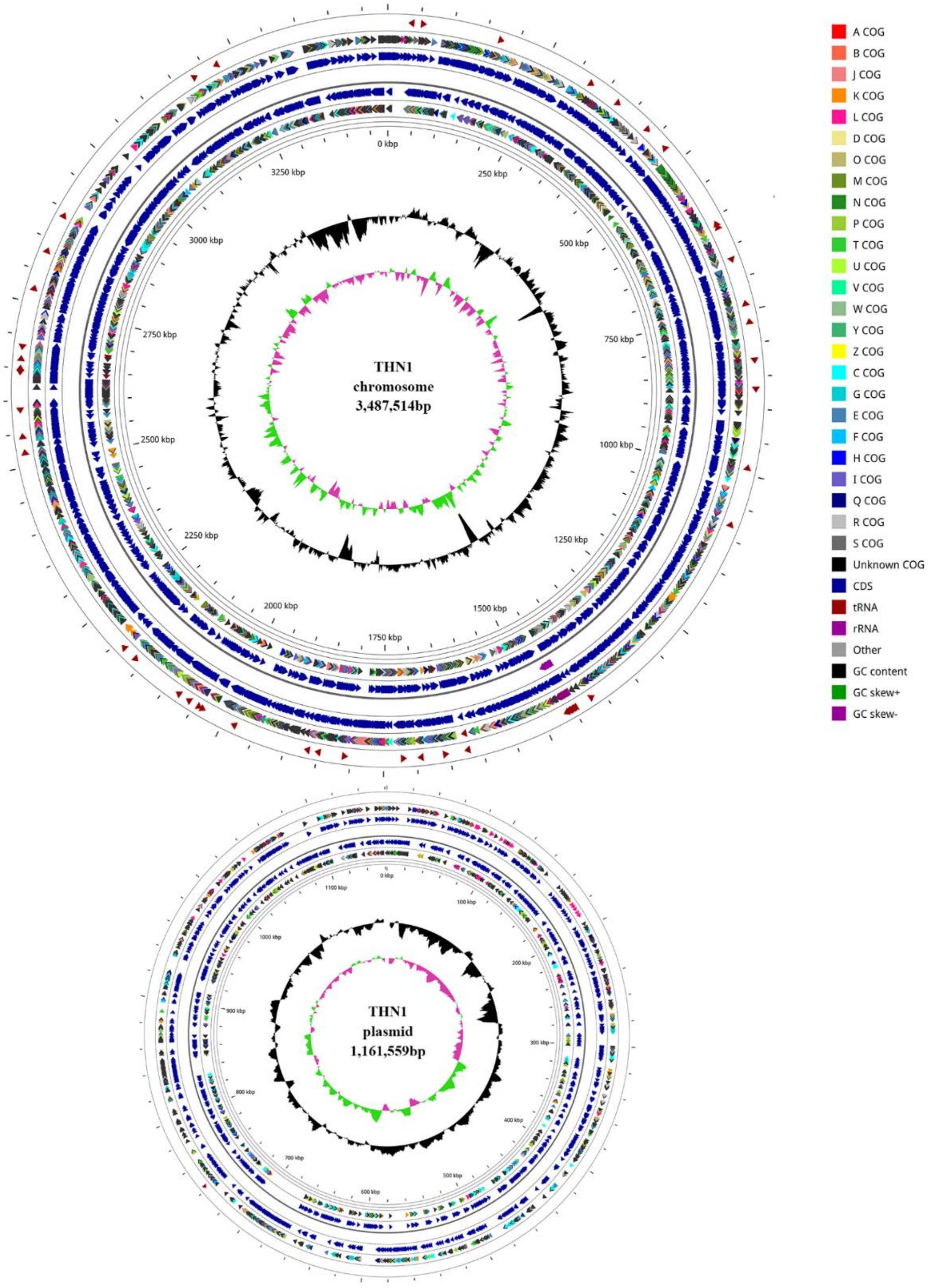

Supplementary Figure S1. **Circular diagrams of the *Novosphingobium* sp. THN1 chromosomes displaying relevant genome features.** The following information is provided from outside to inside: (1) tRNAs (red); (2) assigned COG classes of forward strand protein-coding sequences (CDSs) indicated by colors; (3) forward strand CDSs; (4), rRNAs (purple); (5) reverse strand CDSs; (6) assigned COG classes of reverse strand CDSs indicated by colors; (7) position (in kilobases); (8) G+C content (swell outward/inward indicates higher/lower G+C compared with the average G+C content); (9) G+C skew (purple/yellow indicate positive/negative values). The bar at the top right corner shows the colors used to indicate the functional COG groups.

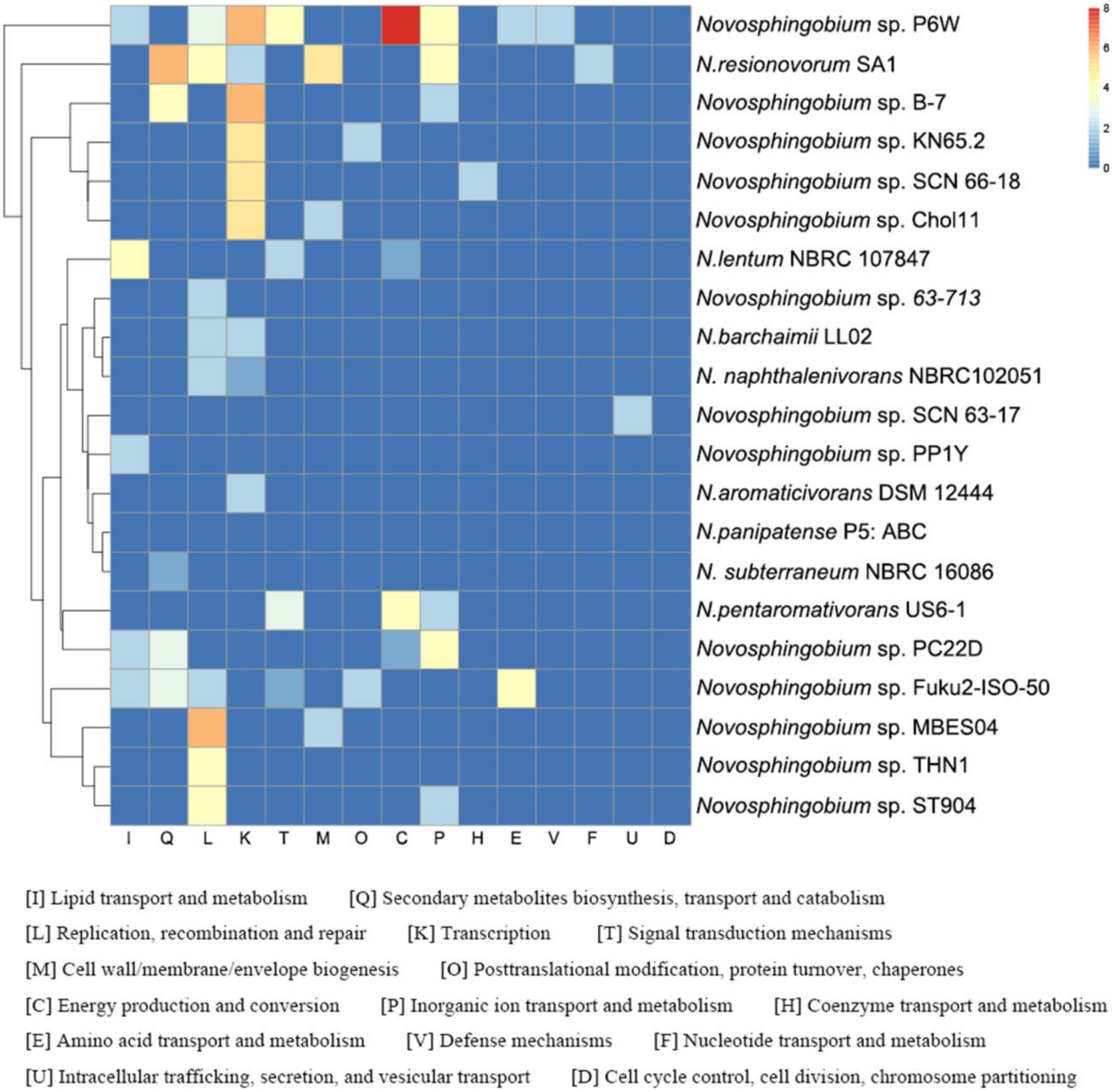

Supplementary Figure S2. A *Novosphingobium* species tree and a heatmap showing the distribution of unique genes annotation based on the clusters of Orthologous Groups (COG).

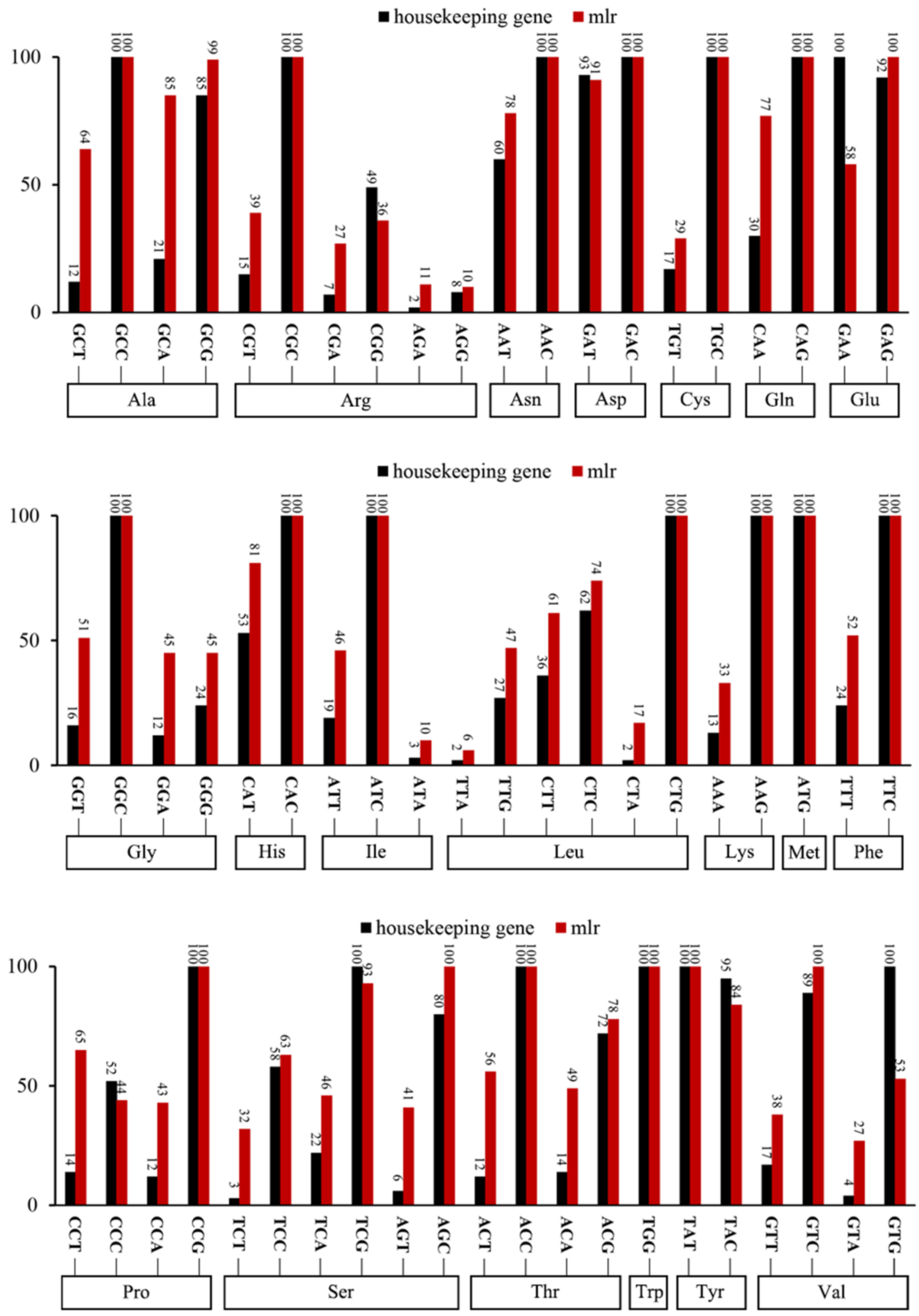

Supplementary Figure S3. The relative adaptiveness values of selected housekeeping genes (black) and *mlr* genes (red) showing the codon usage differences.
